# Inhibitors of the AAA+ Chaperone p97

**DOI:** 10.3390/molecules20023027

**Published:** 2015-02-12

**Authors:** Eli Chapman, Nick Maksim, Fabian de la Cruz, James J. La Clair

**Affiliations:** Department of Pharmacology and Toxicology, College of Pharmacy, University of Arizona, Tucson, AZ 85721-0207, USA; E-Mails: nickmaksim2@gmail.com (N.M.); fabiandlcrz@email.arizona.edu (F.C.); i@xenobe.org (J.J.L.C.)

**Keywords:** p97/VCP/Cdc48, proteostasis, inhibitor, cancer, neurodegeneration, chaperone

## Abstract

It is remarkable that a pathway as ubiquitous as protein quality control can be targeted to treat cancer. Bortezomib, an inhibitor of the proteasome, was first approved by the US Food and Drug Administration (FDA) more than 10 years ago to treat refractory myeloma and later extended to lymphoma. Its use has increased the survival rate of myeloma patients by as much as three years. This success was followed with the recent accelerated approval of the natural product derived proteasome inhibitor carfilzomib (Kyprolis^®^), which is used to treat patients with bortezomib-resistant multiple myeloma. The success of these two drugs has validated protein quality control as a viable target to fight select cancers, but begs the question why are proteasome inhibitors limited to lymphoma and myeloma? More recently, these limitations have encouraged the search for additional targets within the protein quality control system that might offer heightened cancer cell specificity, enhanced clinical utility, a lower rate of resistance, reduced toxicity, and mitigated side effects. One promising target is p97, an ATPase associated with various cellular activities (AAA+) chaperone. p97 figures prominently in protein quality control as well as serving a variety of other cellular functions associated with cancer. More than a decade ago, it was determined that up-regulation of p97 in many forms of cancer correlates with a poor clinical outcome. Since these initial discoveries, a mechanistic explanation for this observation has been partially illuminated, but details are lacking. Understandably, given this clinical correlation, myriad roles within the cell, and its importance in protein quality control, p97 has emerged as a potential therapeutic target. This review provides an overview of efforts towards the discovery of small molecule inhibitors of p97, offering a synopsis of efforts that parallel the excellent reviews that currently exist on p97 structure, function, and physiology.

## 1. Introduction

Over the last decade, genetic and biochemical investigations have revealed the ATPase associated with various cellular activities (AAA+) chaperone p97, also called valosin containing protein (VCP) and Cdc48, as a potential therapeutic target for cancer [1,2,3,4,5,6]. In addition, p97 has been linked both directly and indirectly to neurodegenerative disorders caused by proteostatic malfunction [3,7,8,9,10,11,12,13,14,15,16,17]. This information has led the drive within both academic and industrial laboratories to identify small molecules that can inhibit specific aspects of p97 function [18,19,20,21,22,23,24,25]. While the functional [26,27,28,29,30,31,32] and structural [33,34] aspects of p97 have been reviewed in detail, efforts toward the discovery of small molecule inhibitors of p97 remain predominantly within the primary literature [18,19,20,21,22,23,24,25,35]. The following review provides an overview of the emerging field of small molecule modulation of p97, offering a synopsis of the different structural classes identified to date and provides a brief summary of the biochemical, physiologic, and therapeutic insights gained through these efforts.

## 2. p97 Structure and Function

**Figure 1:**
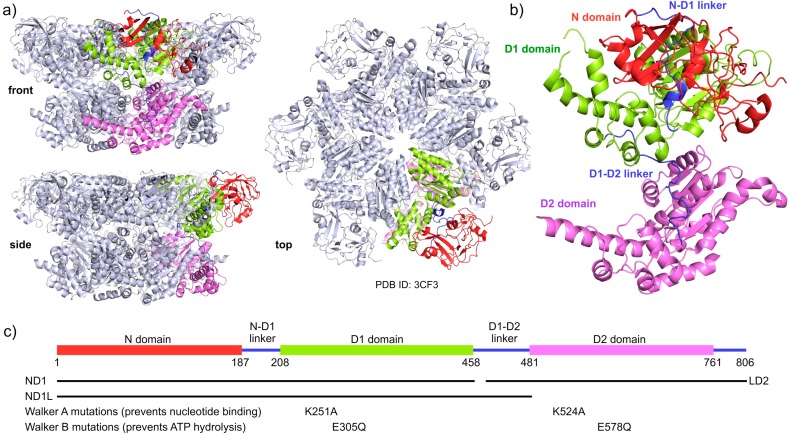
Structure of the AAA+ chaperone p97. (**a**) The structure of the p97 hexamer rendered from PDB ID 3CF3 providing front, side, and top views denoting the positions of the N domain (red), the D1 domain (green), and the D2 domain (magenta) within one subunit; (**b**) Close up of a single subunit illustrating the N domain, D1 domain, and D2 domain along with the N-D1 and D1-D2 linker regions (blue); (**c**) The domain architecture of p97 by amino acid number colored as in a and b (top). A series of deletion and point mutations used by many research groups to dissect the mechanism of p97 inhibitors.

The active form of p97 is comprised of six identical subunits with three domains per subunit and a C-terminal extension (Figure 1a) [33,36,37,38,39,40,41]. The three domains given proximally to distally are: the N-domain, the first AAA domain D1, and the second AAA domain D2 (Figure 1b). The subunits are arranged in a flower shaped structure with a pore running through the center (Figure 1a). The N-domains form the petals of the flower and serve to facilitate cofactor binding and substrate recognition [42,43]. In addition, the N-domains are mobile and may participate in generating the force required for p97 to conduct its physiologic functions [33]. The D1 domain is an ATP binding domain, but early genetic dissections produced some controversy regarding the function of the D1 domains [44,45,46]. Further studies have shown that the D1 domains catalyze the assembly of the hexamer and are the major source of hexamer stability [47]. However, subsequent studies have made it clear that the D1 domains are active ATPases and are coupled to the ATPase activity of the D2-domains [24]. Both the D1 and D2 domains contain the prototypical AAA elements with Walker A and B motifs that allow for genetic dissection of DNA binding and hydrolysis (Figure 1c). The disordered C-terminal extension is the binding site for a variety of cofactors, which can be regulated through C-terminal post-translational modifications [48,49,50].

p97 has been dubbed a “segregase”. This moniker indicates p97 uses the energy of ATP binding and hydrolysis to segregate a protein substrate from another protein, from a protein complex, or from a membrane.

**Figure 2:**
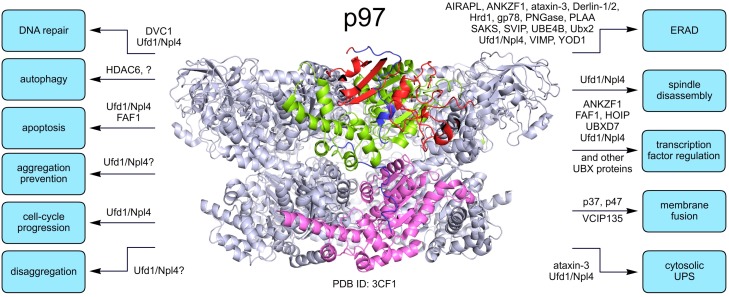
p97 function. The central image is from the highest resolution crystal structure of p97. Two contiguous subunits have been colored by domain: N-domains (red), D1-domains (green), D2-domains (magenta), and the linkers connecting the domains are shown (blue). As demonstrated in the light blue boxes, p97 is involved in a large number of biological processes, which are facilitated by a cohort of cofactors as shown over the arrows.

p97 is one of the most abundant proteins in the eukaryotic cytosol and its segregase function has been linked to a large number of biological processes including endoplasmic reticulum associated degradation (ERAD) [51], mitochondrial associated degradation (MAD) [52], ubiquitin fusion degradation (UFD) [53], homotypic membrane fusion [54], cell cycle regulation [55], autophagy [56], and transcription factor regulation [57,58] (Figure 2). To carry out these diverse functions, p97 employs a large cohort of cofactors (Figure 2), which can be divided into one of three classes: cellular localization factors, substrate recruiting factors, or factors that remodel substrate post-translational modifications [26,27]. The cellular localization factors are often membrane-localized and expose domains on the cytosolic face of a membrane. These cytosolic domains recruit p97 to a site of action. These localization factors are critical to functions such as ERAD and MAD [59,60,61,62,63]. The substrate recruiting factors contain ubiquitin recognition motifs. Although there has been some disagreement, it is generally believed p97 operates on ubiquitylated substrates and that p97 does not have an independent ubiquitin recognition motif. These substrate recruiting cofactors generally bind to the N-domain and recruit ubiquitylated substrates to p97. Finally, p97 has a series of cofactors that alter the post-translational modification state of its clients. These include the addition or removal of ubiquitin [26,27] or the removal of carbohydrates [64].

The best-characterized physiologic function of p97 is the ERAD pathway [51,59,60,61,62]. Here, p97 acts as a force-generating machine to remove misfolded polypeptides from the membrane of the endoplasmic reticulum (ER) for cytosolic, ubiquitin proteasome system (UPS) mediated degradation. p97 is recruited to the ER membrane by the resident cofactor, Ubx2, which exposes an Ubx domain to the cytosolic side of the ER. In turn, the Ubx domain is recognized by the N-domains of p97. The heterodimeric p97 cofactors, Ufd1 and Npl4, act as the substrate recruiting factors, recognizing ubiquitin on the substrate polypeptide to be extracted. p97 then generates a force using ATP binding and hydrolysis to pull the offending polypeptide from the membrane. After extraction, the polypeptide can be recycled through the action of a C-terminal localized cofactor Ufd3 and a deubiquitylase, which prevents degradation. Alternatively, the substrate can be fed to the proteasome facilitated by the action of the E3 (E4) ubiquitin ligase, UBE4B, for destruction [26,27].

## 3. Pathologic Role of p97

It is logical to anticipate that because p97 plays a significant role in regulating a variety of physiologic responses, p97 function will be associated with a number of pathologic states [1,2,3]. More than a decade ago, it was shown that increased levels of p97 in patients with cancer correlated with poor clinical outcomes [3,65,66,67,68,69,70,71]. This was true in many forms of cancer including colorectal carcinoma, pancreatic cancer, thyroid cancer, breast cancer, squamous cell carcinoma, gastric carcinoma, osteosarcoma, and lung cancer. Recent investigations have begun to uncover the reasons why p97 function plays a key role in tumor biology. For instance, investigations on non–small cell lung carcinoma (NSCLC) indicated that p97 was not only up regulated, as was general protein quality control burden, but also that inhibition of p97 led to cell cycle arrest at G_0_/G_1_ [4]. In addition, a direct link was made between p97 and the levels of p53 and NFκB, two transcription factors implicated in cancer cell survival [4]. Finally, recent evidence indicates that tumor cells were susceptible to p97 inhibition [4,20,22,23,72].

Along with its role in cancer, p97 has been implicated both directly and indirectly in neurodegenerative proteinopathies [3,7,8,9,10,11,12,13,14,15,16,17]. Genetic investigations of patients have revealed a series of mutations in p97 that lead to the rare autosomal dominant condition known as inclusion body myopathy with Paget’s disease of bone and frontotemporal dementia (IBMPFD) [73]. In each case, the mutations lead to a catastrophic multi–system collapse that is ultimately lethal [73]. The origin of this pathology remains somewhat enigmatic, however a number of biochemical observations have been made that offer insight. First, in all cases it has been observed that the ATPase rate of the IBMPFD mutants is increased [74,75]. Second, it has been shown that the movement of the N-domains is altered [75,76]. Third, there is an alteration of cofactor binding to the N-domains, which is likely coupled to the alterations in N-domain movement [77]. Additionally, more recent genetic studies have shown a direct link between p97 and amyotrophic lateral sclerosis (ALS) [78]. Indirectly, p97 has been shown to be associated with aggregates resulting from Alzheimer’s disease, Huntington’s disease, Machado–Joseph disease, prion diseases, Parkinson’s disease, and a variety of tauopathies [3,7,8,9,10,11,12,13,14,15,16,17]. It is however unlikely that in this context, inhibition of p97 is the desired means of treatment, in fact, restoration of p97 function would be desired.

## 4. Protein Quality Control as a Drug Target

Although largely seen as an unlikely method to treat cancers or any other disease at the time, targeting protein quality control has emerged as a valid clinical entry [79]. The pioneering efforts that brought the proteasome inhibitor, bortezomib, to the clinic were a remarkable drug discovery success story [79]. Although the precise mode by which bortezomib exerts its selective function remains controversial, evidence suggests a key role for the secretory pathway. This has been proposed to be the reason proteasome targeted therapies are effective at treating blood cancers, such as mantle cell lymphoma and multiple myeloma, that derive from cells that produce large numbers of secretory proteins. Although bortezomib has seen success, lack of generality and a number of problems have prompted researchers to continue investigations into targeting protein quality control. This bore fruit with the expedited FDA approval of carfilzomib in July 2012 to treat bortezomib-resistant myelomas. Presently, further clinical trials are underway to evaluate carfilzomib in other clinical contexts with hope that it will be effective at treating other malignancies, including cancers that are not susceptible to bortezomib treatment. The successes of bortezomib and carfilzomib also argue that targeting protein quality control in general might be a viable treatment option, which has prompted investigators in both academic and industrial settings to expand the search for other targets in the protein quality control pathway, including p97.

As stated above, p97 is a highly abundant cytosolic protein that is involved in numerous cellular functions. As outlined in Figure 2 there are many possible pathways that might be affected by the action of a p97 inhibitor. Many of these pathways represent proven therapeutic options for the treatment of cancer [80,81,82,83,84,85]. One area of particular interest is the ERAD pathway [59,60,61,62]. Cells that produce an abundance of secreted proteins, such as B-cells, have a naturally elevated burden on their secretory pathways. This may very well be a tipping point for therapeutic intervention. In addition, p97 activates pro–survival transcription factors, bulk protein destruction (autophagy) and regulates the cell cycle, all of which are proven to be important in cancer therapy [1,2,3,4,5,6,80,81,82,83,84,85]. The following sections provide an overview of a series of therapeutic leads. This discussion serves not only to highlight the key design components within the lead discovery effort, but also, and perhaps more importantly, to illustrate how the union between lead discovery and target validation plays a key role in clinical translation.

## 5. Eeyarestatin I

**Figure 3:**
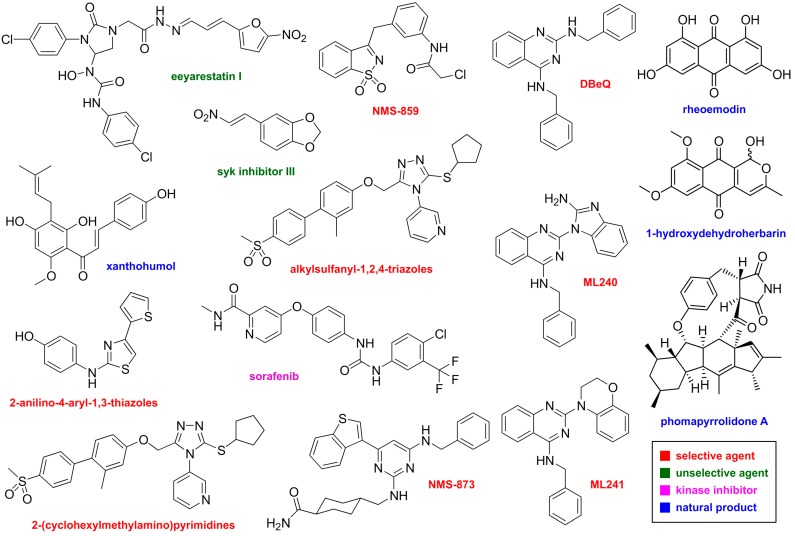
Structures of the current collection of p97 inhibitors. Compounds can be sorted by classes as given by unselective agents (green), selective agents (red), natural products (blue) and a kinase inhibitor that causes misregulation of p97 by blocking phosphorylation of the C-terminus (violet; not discussed in text) [90].

In 2004, a team at Harvard Medical School and Massachusetts General Hospital developed a fluorescence protocol using the class I major histocompatibility complex (MHC) heavy chain linked to enhanced green fluorescent protein (EGFP) to assay for molecules that block ERAD function [86]. In this assay, stabilization of the fluorescent signal indicated inhibition of some part of the ERAD pathway. Using this procedure in a high throughput format led to the discovery of eeyarestatins I (Figure 3) and II (EerI and EerII). Subsequent in depth evaluation demonstrated that EerI inhibited dislocation of the reporter from the ER membrane, but did not inhibit the proteasome, offering a molecule with a novel mode of action (MOA) [87]. EerI was found to affect ERAD by interfering with the p97-associated deubiquitylating enzyme ataxin-3 [18]. A follow-up experiment using major histocompatibility complex (MHC) protein demonstrated that EerI had a relatively low potency with IC_50_ values of ~70 µM for inhibition of substrate translocation [18]. Similar results were observed when mammalian cells bearing the t–cell receptor α (TCRα)–GFP reporter protein were treated with 10 µM EerI [88]. EerI has also been shown to inhibit Sec61–mediated protein translocation [88]. *In vitro*, EerI inhibited translocation and subsequent *N*-glycosylation of the P2X2 purinergic receptor in a dose-dependent manner [88]. EerI may affect other target proteins, as evidenced by its disruptive effects between ribosome-nascent chain complexes and the translocon Sec61, although EerI’s exact function in this pathway is unclear [88].

Biochemically, EerI has been shown to have two active domains: an aromatic domain that binds to membranes and a nitrofuran moiety, which inhibits p97 [87]. Surface plasmon resonance (SPR) data confirm that EerI binds directly to p97 with a K_d_ of 5–10 µM [87]. It has been suggested that the difference between cellular and *in vitro* models may result from metabolism of EerI [18]. As a test of reversibility, cells expressing an Ub^G76V^GFP reporter were used. This Ub^G76V^GFP reporter used an in-line ubiquitin fusion with a G76V mutation at its C-terminus. The G76V mutation prevents hydrolysis of the ubiquitin fusion by deubiquitylating enzymes. This reporter system is a well-established strategy in probing the ubiquitin proteasome system [89]. The reporter cells were treated with EerI, washed extensively to remove unbound molecule, and then degradation of the UbG76VGFP substrate monitored over 4 h. Even after removal from cell culture, EerI continued to prevent GFP degradation, suggesting that it irreversibly binds to p97 [87].

As alluded to above, EerI-mediated inhibition of both p97 and other ER-related functions results in several downstream effects. In EerI treated cells, the rise in misfolded proteins in the ER leads to the unfolded protein response (UPR), which, if uncorrected, results in activation of apoptotic programs [91]. The UPR is a cellular response designed to keep tight control of the secretory pathway by increasing levels of ER resident chaperones and decreasing overall protein synthesis as needed to meet cellular demands [92,93]. The UPR is also activated as a consequence of ERAD inhibition. p97 levels have long been known to correlate negatively with clinical outcome in cancer patients, but the use of EerI was critical in dissecting the relationship between cancer and p97 function [4]. In particular, the treatment of cancer cells with EerI showed that p97 inhibition restored p53 and NFκB levels, established cancer markers. This work also led investigators to examine the synergy between p97 and proteasome inhibition. EerI has been shown to work in concert with bortezomib in inducing ER stress-mediated apoptosis in cancer cells [94,95]. One study demonstrated that these compounds worked in tandem to simultaneously promote NOXA expression while inhibiting H2A-ubiquitylation, a NOXA antagonist [94]. A second study showed a combination of bortezomib and EerI led to a catastrophic disruption of the secretory pathway and increased apoptotic cell death [95]. A more recent study demonstrated that these two compounds effectively induced ER-mediated apoptosis in cervical cancer cells by up regulating the pro-apoptotic protein CCAAT-enhancer-binding protein homologous protein (CHOP) [96].

EerI is an important lead due to its modular architecture, its effects on the ERAD pathway, and its synergistic role with bortezomib. However, EerI suffers from a poorly understood mechanism, irreversibility, and low potency. Initial investigations have begun to simplify the aromatic domain, giving rise to derivatives with increased potential [97]. In spite of these liabilities, EerI has been a powerful tool in the elucidation of p97 cellular function. It has been used to demonstrate that the type II deiodinase is retrotranslocated in a p97-mediated fashion. This function is critical to thyroid hormone regulation [98]. Additionally, EerI was used to demonstrate the connexin50 mutant, CX50fs, that causes cataracts is degraded through the ERAD pathway in a p97 dependent manner [99]. This has important implications for the treatment of degenerative eye diseases. Finally, EerI was used to establish a critical link between the lysosomal storage disease, Tay-Sachs disease, and ERAD. In particular, inhibition of ERAD was shown to increase folding of lysosomal proteins through decreased degradation rates, suggesting new avenues for treatment [100]. Combined these studies illustrate the power of small molecule inhibitors as cell biological tools, especially in the context of a protein such as p97 with diverse cellular functions and a complex pathology.

## 6. 2-Anilino-4-aryl-1,3-thiazoles

The first targeted discovery of p97 inhibitors came from a high-throughput screening effort using a glucokinase coupled ATPase assay [19]. This effort revealed 2-anilino-4-aryl-1,3-thiazole as a lead scaffold. Medicinal chemistry efforts using a Hantszsh thiazole synthesis produced a series structure-activity relationships (SAR) and these data were useful in identifying a set of p97 inhibitors with nanomolar IC_50_ values. The resulting lead molecules were also evaluated in a cellular assay that made use of an Ub^G76V^-luciferase reporter. In this system, if p97 or other members of the UPS are inhibited, the degradation of luciferase is blocked leading to an accumulation of the reporter protein and increased chemoluminescence in the presence of luciferin. This collective produced a molecule, 2-anilino-4-aryl-1,3-thiazole-2-thiophene (Figure 3) that demonstrated potent activity, IC_50_ value of 110 nM, *versus* p97 ATPase activity along with an EC_50_ value of 90 nM in the cellular Ub^G76V^-luciferase reporter assay. Although an important proof of concept, the molecules were not thoroughly evaluated for specificity and were later shown to interfere with other aspects of the UPS, therefore restricting further development [101].

## 7. Syk Inhibitor III

The initial efforts that identified EerI and 2-anilino-4-aryl-1,3-thiazole-2-thiophene demonstrated the power of p97 inhibitors in cell biology, their therapeutic potential, and the possibility of finding potent small molecule p97 inhibitors. However, previous studies were rife with complicating factors. In an effort to streamline this process and to discover more potent and, more importantly, specific molecules, a team led by Deshaies at the California Institute of Technology introduced a series of cellular assays to further refine the correlation between compound potency and p97 selectivity [102]. Since p97 is involved in the general protein quality control pathway, it is essential to differentiate between general UPS and p97 specific functions. To facilitate this, the known UFD reporter Ub^G76V^GFP was co-expressed with the p97-independent degradation domain of hypoxia inducible factor 1α (HIF1α) fused to luciferase (ODD-Luc). In parallel, cells were developed as controls that contained both the Ub^G76V^GFP with the ubiquitin independent degradation domain of ornithine decarboxylase (Luc-ODC) and Ub^G76V^GFP co-expressed with luciferase [102]. Using these screens in parallel, 3,4-methylenedioxy-β-nitrostyrene, also known as Syk inhibitor III, was identified by its selective activity in the Ub^G76V^GFP reporter system relative to the luciferase reporters, indicating this molecule operated predominantly in the p97-dependent pathway. In addition, the authors explored a series of molecules known to inhibit various aspects of the UPS including E1 and E3 inhibitors and found that the collective of assays reported as expected.

## 8. Quinazolines

In 2011, further efforts led by Ray Deshaies described the discovery of *N^2^,N^4^*-dibenzylquinazoline-2,4-diamine (DBeQ) as the first reversible, selective small molecule p97 inhibitor (Figure 3) [20]. DBeQ was discovered by using an ATPase assay in a high-throughput screen of the NIH Molecular Libraries Small Molecule Repository [20]. Initial studies focused on addressing the aforementioned shortcomings of previous p97 inhibitors. Characterization of DBeQ demonstrated strong inhibition against both wild type p97 and mutants lacking the active site cysteine residues (indicating the interaction was likely not covalent). In addition, DBeQ showed potent inhibition of p97 mediated Ub^G76V^GFP degradation. Similar to the efforts described above on the Syk inhibitor III, the team found that DBeQ showed greatly diminished inhibitory effects when assayed against ODD-Luc and Luc-ODC, p97-independent degradation pathways, indicating p97-specificity. Further investigation indicated that DBeQ’s quinazoline core, a known protein kinase inhibitor, did not significantly inhibit any of the 170 kinases that were evaluated. DBeQ also showed reversibility *in vivo*, with Ub^G76V^GFP degradation returning after removing the DBeQ from the cell culture by washing. Lineweaver–Burke analysis was used to argue DBeQ competitively inhibited p97, however, given the cooperative nature of p97 [103], a more complex analysis is required to truly determine the mechanism of inhibition [104]. Although a detailed discussion of this is outside the scope of this review, in general inhibitors of this sort would be better referred to as K- or V-type inhibitors. V-type inhibitors affect the k_cat_ and K-type inhibitors affect the K_d_. It is more accurate to assess inhibition in the context of the principles of allosteric linkage, but for a large multi-subunit enzyme such as p97 this is a daunting task. In general, a detailed analysis of the binding of both the inhibitor and the substrate is required and then a detailed analysis of how these events are coupled. In the present case it is impossible to conclude the inhibitor competes for the same binding site as ATP. It could just as easily bind to an ATP binding site in another subunit or in the other pocket of the same subunit and send an allosteric signal blocking binding to a neighboring subunit [104,105,106,107]. Further argument against a true competitive mechanism arose through later studies that showed that DBeQ more strongly inhibited p97-K251A, with an unaltered D2 domain, at higher ATP levels, suggesting that its’ binding site may lie elsewhere or it may require ATP bound in D1 to properly inhibit [101].

As part of their studies, the Deshaies team also evaluated DBeQ for its effect on protein degradation pathways that require p97 for processing. The TCRα-GFP reporter was used to examine the effects on the ERAD pathway. An increase of fluorescence (indicating inhibition of the ERAD pathway) was detected when either p97 or the proteasome was inhibited. Treatment with DBeQ increased the level of fluorescence in a manner similar to the proteasome inhibitor MG132 (a positive control) and did not appear using vehicle (negative control).

Next the team inspected the effects of DBeQ on UPR. As described above, the UPR is also activated as a consequence of ERAD inhibition. If there is a protein quality control problem in the secretory pathway and it fails to be corrected, UPR leads to an up-regulation CHOP, which signals ER stress-induced apoptosis in part by down regulating p21. HeLa cells treated with DBeQ showed a dose-dependent increase in CHOP levels with no increase in p21, indicating that DBeQ had inhibited ERAD and activated the UPR to counteract this effect, a response that ultimately activates apoptotic pathways.

In addition to the ERAD and UPS arms of protein quality control, p97 has been shown to interact with the autophagic arm of proteostasis. While the mechanism of these events has not yet been fully resolved, p97 has been shown to be required for autophagosome maturation [56]. DBeQ was also shown to impair autophagic protein degradation by inhibiting the fusion of autophagosomes with lysosomes. This process is measured by looking at the levels of LC3-II, which is normally degraded by autolysosomes, but becomes stabilized when autophagy is compromised. Treatment with DBeQ stabilized LC3-II from degradation, whereas treatment with vehicle did not.

As mentioned above, DBeQ leads to a compromise of ERAD, which induces the UPR and increases levels of CHOP, implying apoptosis should be initiated. This mode of cell death was more thoroughly examined by looking at traditional apoptotic markers. Here, the Deshaies’ team followed up on early indications that DBeQ was more effective at inducing apoptosis in cancer cells than their noncancerous counterparts. Both p97 RNAi–depleted and DBeQ treated HeLa cells showed a marked increase in caspase–3 and caspase–7 activation, with DBeQ favoring the caspase-9 apoptotic pathway over the extrinsic caspase–8 pathway. This increase in apoptotic factors led to a corresponding decrease in cell viability. Interestingly, DBeQ showed a greater short-term inhibitor capacity compared to MG-132, which typically has shown enhanced cell growth inhibition (10–20 fold greater than DBeQ).

In an effort to improve on DBeQ, the quinazoline scaffold was used as a starting point for medicinal chemical advancement [22]. Using the same battery of assays described above as a guide, two new inhibitors, Pubchem CID49830258 (ML240) and Pubchem CID49830260 (ML241) were developed with improved specificity and potency (Figure 3). All three leads, ML240, ML241, and DBeQ, displayed similar inhibitor activities when screened against ERAD. Western blot analyses indicated that both ML240 and ML241 increased the level of a TCRα-GFP reporter when compared to vehicle. Of note, ML240 resulted in a higher accumulation of TCRα-GFP compared to ML241 in both the nuclear plus membrane and insoluble fractions. These results also indicated the accumulation of wild type cystic fibrosis transmembrane conductance regulator (CFTR) and the F508Δ mutant, proteins normally rapidly degraded in ERAD, indicating that both ML240 and ML241 inhibit p97’s ERAD function.

Despite their similar characterization and effect on the ERAD pathway, ML240 and ML241 exhibited different effects on the autophagy and apoptotic pathways. ML240, but not ML241, caused an accumulation of LC3-II, indicative of an impaired autophagic pathway. ML240 was significantly more potent (24–70 fold) in blocking cell proliferation. Western blot analysis revealed ML240 led to cleavage of the caspase substrate poly-(ADP-ribose) polymerase (PARP), suggesting that ML240 acted through an apoptotic process, while ML241 did not. The rapid accumulation of LC3-II and presence of PARP instigated further analysis of ML240. Upon addition of ML240, cells quickly (*t* < 10 min) accumulated activating transcription factor 4 (ATF4), signifying an almost immediate activation of the UPR. Additionally, ML240 induced caspases-3 and caspase-7 more efficiently than DBeQ, the authors suggested this was independent of the initiator caspases caspase-8 and caspase-9. This conclusion was based on experiments using Jurkat cells without caspase-8 or caspase-9, however, it remains possible one compensates for the other as the double knockout was not used. These findings provide a more convincing argument for ML240 as a starting point for therapeutic consideration. However, it is equally interesting to note that ML241’s ERAD-specific inhibition opens the possibility of developing pathway specific inhibitors, which could be useful in both elucidating p97’s role in cellular pathways, as well as developing pathway-specific therapies. 

Later biochemical studies began to identify the differential effects of DBeQ, ML240, and ML241 in cellular models. For instance, Chou’s team found that ML240 and ML241 selectively target the D2 domain. These studies were conducted using a series of Walker A and B mutants (Figure 1c) and a truncated version of p97 housing the N-domains, the D1-domains and the D1-D2 linker (this construct was named ND1L). Using similar methods they also found that DBeQ targeted both domains, therein providing an initial explanation of the previously observed selectivity. In addition, some of the IBMPFD mutations decreased the potency of ML240 and ML241, which is an interesting result, given these mutations are thought to change the off rate of ADP in the D1 domain and not to necessarily affect the D2 ATPase activity [101]. Finally, these studies revealed differential effects in the presence of the p97 cofactor, p47, which is the prominent cofactor in homotypic membrane fusion. In particular, DBeQ was much less affected by the presence of p47 than the other quinazolines evaluated in their studies. These data collectively argue for the possibility of tuning small molecules to specific p97 physiologic functions to treat a specified pathologic state as well as generating specificity in the context of malignancies, both very exciting propositions [24].

Most recently, the Chou team examined the use of their collective of mutants as the primary screening entity to identify leads with improved selectivity for a given p97 domain [24]. In these studies, the team prepared a panel of 200 quinazolines and screened for their inhibition of the D1 or D2 domains of p97. From this effort, compounds selective for either the D1-domain or the D2-domain were discovered. Excitingly, the team also discovered materials whose selectivity increased in the presence of the cofactor p47. Overall, these studies not only validated p97 as a target but also highlighted the potential of developing p97 inhibitors tuned to a particular physiologic function.

## 9. Alkylsulfanyl-1,2,4-triazoles

In a high-throughput screening campaign a team of scientists at Nerviano Medical Sciences and Genentech discovered an alkylsulfanyltriazole to be a moderate, allosteric p97 inhibitor [21]. Extensive medicinal chemistry and SAR studies revealed a series of improved compounds that displayed sub-micromolar IC_50_ values. In addition, the top leads were shown to increase the levels of ubiquitylated proteins, cyclin E, and CHOP in a dose-dependent manner *versus* a vehicle control. Total ubiquitylated proteins are expected to go up upon p97 inhibition because many membrane bound and cytosolic proteins require p97 chaperoning to reach the proteasome. All three compounds tested showed a dose dependent increase in the amount of total ubiquitylated proteins. The level of the cell-cycle protein, cyclin E, also increased in a dose-dependent manner, indicating the tested molecules influence the cell cycle, however this was not extensively investigated. Finally, the ER stress related protein, CCAAT-enhancer-binding protein homologous protein (CHOP), was examined. Again, CHOP showed a dose-dependent increase in protein level, indicating activation of the unfolded protein response pathway. In this study, interference with other parts of the UPS was not ruled out, so cellular p97 specificity cannot be assured.

However, these studies provided a foundation for a second generation of lead discovery, resulting in both covalent inhibitors and allosteric inhibitors [23]. In these extremely detailed studies, the authors began by carefully analyzing the effects of p97 knockdown using siRNA to establish critical biomarkers for p97 inhibition. To determine cell-line specific effects, the authors used three different cell lines from three types of cancer: a cervical carcinoma line (HeLa); an osteosarcoma line (U2OS); and a colon adenocarcinoma line (HCT116). In each of the three, total ubiquitylated proteins were increased; cyclin E was increased; GRP78, CHOP, and GADD-34 were increased (markers of ER stress); LC3B levels increased (indicating compromised autophagy); and caspase 3 increased with concomitant PARP cleavage (indicating apoptosis). The specific effects on the cell cycle were also examined and indicated cell-type differences with HeLa cells arresting in the G1 phase, U2OS cells showing no cell cycle effects, and HCT116 cells arresting in the G2-M phase. Finally, a Bliss analysis was carried out to look at the synergy between p97 knockdown and FDA approved cancer drugs. This analysis demonstrated synergy between p97 inhibition and DNA-damaging agents, antimitotics, and stress-inducing agents.

With these data in hand and an established high-throughput screening platform, one million compounds were screened. From this screen, three classes of compounds were chosen for further investigation and elaboration. The first compound class was from a covalent inhibitor, NMS-859 (Figure 3). This compound was shown to bind to Cys522 in the D2 ATP binding pocket. The second class was from a modest allosteric inhibitor (NMS-862). The third class, were compounds that showed IC_50_ shifts as a function of ATP concentration, indicating a possible competition for the ATP pocket, although this was not elaborated. The covalent compound showed a submicromolar, time-dependent IC_50_ value against p97 and single digit IC_50_ against HCT116 and HeLa cells. The allosteric molecule showed a low micromolar IC_50_ but had no activity against the two cell lines. Importantly, both the covalent and allosteric molecules showed no inhibition of other ATP-utilizing enzymes including VPS4B, RuvBL1, NSF, SPATA5, HSP90, or 53 kinases. Finally, the ATP-sensitive molecules had low micromolar IC_50_s in both biochemical and cellular assays, but because of off-target effects, as assessed by a series of biochemical assays against other ATP utilizing enzymes, these were abandoned and will not be discussed further here.

Before going into a detailed assessment of the activities of the various inhibitors, the authors very eloquently mapped the binding site for the allosteric inhibitor class and proposed an interesting and very plausible mechanism of action. Because p97, was resistant to their efforts at getting a co-crystal structure, the authors used a pair of inhibitor derivatives harboring arylazides for photo-affinity labeling. Cross-linking coupled with peptidase digestion and mass spectrometry mapped to Lys615 and Lys616. These two residues are in a tunnel between the D1 and D2 domains. To further assess this as the binding site, several residues in this general region were mutated and some of these mutations were found to completely block inhibition by NMS-873 or to cause a large increase in the IC_50_, arguing for this tunnel as the bona fide binding site. A series of ADP competition experiments were also carried out and it was found the compounds actually increased the K_d_ for ADP. This led to a model in which the molecules interact with the intersubunit signaling motif, preventing the required shift of the arginine fingers, which freezes the D2 domain in the ADP-bound state.

Next, employing the data from the reported genetic experiments, the cellular effects of the covalent molecule (NMS-859) and the best allosteric molecule (NMS-873) were examined extensively using HCT116 cells as a model. Both molecules faithfully recapitulated the genetic data showing increases in all of the marker proteins, induction of UPR, compromised autophagy, and apoptosis. In addition, the compounds arrested the cells in the G2 phase. These data were further confirmed using fluorescence microscopy and high-content screening.

These inhibitors were also compared to the proteasome inhibitor bortezomib. First, because proteasome inhibitors are known to lead to aggresome formation, this was examined and the p97 inhibitors were found to not form aggresomes. Finally, a panel of 37 cancer cell lines was treated with NMS-873 or bortezomib and very different results were observed with specific cell lines being differentially sensitive to the two inhibitors, arguing for p97 as a molecular protein quality control target that will have therapeutic use beyond blood cancers.

Finally, a recent study by Chou and coworkers further explored the mechanism and specificity of NMS-873 [101]. These researchers looked at a series of p97 mutants including Walker A and B mutations in D1 and D2 and truncation mutants (the N- and D1-domains and the N- and D1-domains plus the linker between D1 and D2). In addition, the effect of the presence of the homotypic membrane fusion cofactor, p47, which inhibits ATPase activity, was examined. These investigations confirmed ATP-insensitivity and showed the activity of NMS-873 was only affected by the D2 Walker B mutation. These mutant studies showed that in addition to the mechanism previously proposed, NMS-873 also prevented ADP release from the D1 domain. Finally, the authors discovered that the presence of p47 also increased the IC_50_ of NMS-873. These studies added to the mechanistic understanding of this potent allosteric modulator, confirmed the high specificity of NMS-873, and made the important discovery that it might be possible to discover molecules to target specific p97 activities.

## 10. Xanthohumol

Synthetic compounds have not been the only materials identified as p97 inhibitors. The natural product xanthohumol (Figure 3), which is present in hops (*Humulus lupulus*), was discovered in 2012 to inhibit autophagy [108]. In an effort to understand its MOA, the authors used a xanthohumol affinity resin and were able to capture p97. The team then used a series of truncated mutants to demonstrate that xanthohumol bound to the N-domains of p97. The authors then compared the activity of xanthohumol to p97 siRNA to identify common cell physiologic effects and specific autophagic responses. Although specificity was not extensively explored, these studies demonstrated that xanthohumol partially induced autophagy and upregulated CHOP in a p97-dependent manner.

## 11. Natural Product p97 Inhibitors from Functional Chromatography

In a recent investigation using a technique referred to as functional chromatography [109], our laboratories isolated three fungal derived natural products with distinct mechanisms of action that inhibited p97 activity [25]. Functional chromatography uses a protein, in this case p97, linked to a solid support as a vehicle to purify molecules from crude mixtures such as natural product extracts. The mechanistic differences likely came about because there is not inherent biochemical bias in functional chromatography. The three molecules isolated were revealed to be rheoemodin, 1-hydroxydehydroherbarin, and phomapyrrolidone A (Figure 3). Limited mechanistic investigations showed that rheoemodin was ATP-sensitive, whereas the other two molecules were not. In addition, the activity of rheoemodin was lost when the cysteines were removed from p97, whereas the other two molecules did not lose activity in this context. Further investigation of this led to the observation the rheoemodin required the cysteine in D1 to be intact, arguing for a D1 selective mechanism of action. We also evaluated the inhibition of the N-D1-L truncation mutant and found rheoemodin to inhibit this construct to the same degree as wild-type p97 (unpublished results).

The precise mechanisms of action for these molecules remain to be illuminated, but each of the compounds showed biochemical specificity relative to other ATPases (GroEL, ClpX, and NSF) and they also showed good effect in a series of cell–based assays, including specificity for p97 relative to the proteasome or other UPS components. Addition of each of the molecules to cells expressing Ub^G76V^GFP (a measure of p97 mediated UPS) or TCRα-GFP (a measure of p97 dependent ERAD) but not HA-CD3δ (a measure of p97 independent ERAD) led to a dose dependent increase in the reporter molecule. In addition, the three natural products led to a dose dependent increase in UPR, LC3B levels, and ultimately apoptosis. These studies not only provided a new approach to identify p97 inhibitors, but clearly demonstrated the immediate need to identify the key biochemical features in p97 modulation that are vital for the anti-tumor and anti-cancer applications.

## 12. 2-(Cyclohexylmethylamino)pyrimidines

While this manuscript was being revised, an extensive medicinal chemistry effort was conducted to develop a hit compound, 2-alkylsulfanylpyrimidine that hailed from a screen of one million compounds [72]. 2-alkylsulfanylpyrimidine had an IC_50_ value of 4.8 µM against p97 ATPase activity, but showed no activity against HCT116 cells. The researchers argued this might be due to the presence of a carboxylic acid moiety preventing crossing of the cell membrane, so they opted to carry out an extensive medicinal chemistry campaign. Initial attempts to obtain a co-crystal structure failed, eliminating the possibility of structure-based design, so a ligand-based approach was deployed. To do this, two primary synthetic strategies were used: chlorinated pyrimidines were coupled to fragments using metal catalyzed reactions and the pyrimidine ring was synthesized using the condensation of acetoacetates with functionalized guanidines. A total of ~100 molecules was synthesized and tested to produce a molecule (Figure 3) with 65-fold more potency and a low micromolar IC_50_ value against HCT116 cells. In addition, this compound showed a dose-dependent increase in poly-ubiquitylated proteins and the UPR reporter, CHOP.

**Table 1:** Compounds discussed in the present review.

Compound	Mechanism	p97 IC_50_ (µM)	Selectivity	Cellular
Eeyarestatin I	Allosteric	5–10 (K_d_)	Does not stabilize ODD-Luc or Luc-ODC	Ub^G76V^GFP IC_50_ 3.7 µM
2-anilino-4-aryl-1,3-thiazole-2-thiophene	ATP-sensitive	0.11	Not reported	Ub-Luc stabilization EC_50_ 0.09 µM
Syk inhibitor III	Covalent; modifies D2 ATPase pocket	1.7	ODD-Luc IC_50_ 5.9 µM Luc-ODC IC_50_ > 30 µM	Ub^G76V^GFP IC_50_ 1.6 µM
Alkylsulfanyl-1,2-4-triazoles	Allosteric	0.063		IC_50_ 0.38 (HCT116 cells)
DBeQ	ATP-sensitive; binds D1 and D2	2.6	ODD-Luc IC_50_ 56 µM Luc-ODC IC_50_ 45 µM	Ub^G76V^GFP IC_50_ 2.3 µM Extensive cellular studies
ML-240	ATP-sensitive; D2 selective	0.11	ODD-Luc IC_50_ 28 µM	Ub^G76V^GFP IC_50_ 0.9 µM Extensive cellular studies
ML-241	ATP-sensitive; D2 selective	0.11	ODD-Luc IC_50_ 46 µM	Ub^G76V^GFP IC_50_ 3.5 µM Extensive cellular studies
NMS-859	Covalent; binds D2 ATP pocket	0.37	NSF, SPATA5, VPS4B, RuvBL1, HSP90, 50 kinases > 10 µM	Extensive cellular studies; toxicity and p97 specific pathways
NMS-873	Allosteric; D1-D2 interface	0.02	NSF, SPATA5, VPS4B, RuvBL1, HSP90, 50 kinases > 10 µM	Extensive cellular studies; toxicity and p97 specific pathways
Xanthohumol	Binds to N-domains	Not reported	Not reported	Examined ERAD, UPR, and autophagy
Rheoemodin	ATP-sensitive; D1 selective	39.8	NSF, ClpX, GroEL > 200 µM; no protection of CD3δ	Ub^G76V^GFP and TCRα stabilized, CD3δ not stabilized, poly-Ub increased, UPR activated, autophagy inhibited, apoptosis activated
1–Hydroxydehydroherbarin	Allosteric; unknown binding site	21.7	NSF, GroEL > 200 µM; no protection of CD3δ	Ub^G76V^GFP and TCRα stabilized, CD3δ not stabilized, poly-Ub increased, UPR activated, autophagy inhibited, apoptosis activated
Phomapyrrolidone A	Allosteric; unknown binding site	6.6	NSF, GroEL > 200 µM; no protection of CD3δ	Ub^G76V^GFP and TCRα stabilized, CD3δ not stabilized, poly-Ub increased, UPR activated, autophagy inhibited, apoptosis activated
2-(Cyclohexyl-methylamino)pyrimidine	ATP-sensitive; unknown binding site	0.074 µM	NSF, SPATA5, VPS4B, RuvBL1, HSP90, 50 kinases > 10 µM	IC_50_ 5.82 (HCT116); poly-Ub increased; UPR increased

## 13. Conclusions

While early in the pipeline, p97 represents a novel and exciting target for the treatment of cancer and perhaps other afflictions. Recent discoveries of natural and synthetic p97 inhibitors have helped to validate p97 as a target (Table 1). In parallel, these studies have facilitated a number of key cellular investigations, and have identified the salient mechanistic features required for translating potential therapeutic leads for p97. In addition, a number of biochemical and structural investigations [26,27,28,29,30,31,32,33,34] along with novel discovery approaches [25] have shown that p97 has multiple binding sites and potential mechanisms of regulation. Importantly, these differential mechanisms have suggested the potential of modulating specific p97-dependent physiologic functions in cells or perhaps even *in vivo*. This exciting possibility may provide a means of developing a better mechanistic understanding of the many pathways p97 regulates. Recent efforts from Cleave Bioscience have also led to the development of the first molecule to enter clinical trials. This molecule, CB-5083, was developed using the ML240 scaffold as a lead [35]. In harmony with drug discovery, the leads identified within these programs also serve as key mechanistic probes that not only provide vital tools to unwind the complex networks regulated by p97 but also offer materials to decode the unique interplay associated with proteostasis.
