# Correction: Chapman, E.; *et al*. Inhibitors of the AAA+ Chaperone p97. *Molecules* 2015, *20*, 3027-3049

**DOI:** 10.3390/molecules20034357

**Published:** 2015-03-09

**Authors:** Eli Chapman, Nick Maksim, Fabian de la Cruz, James J. La Clair

**Affiliations:** Department of Pharmacology and Toxicology, College of Pharmacy, University of Arizona, Tucson, AZ 85721-0207, USA; E-Mails: nickmaksim2@gmail.com (N.M.); fabiandlcrz@email.arizona.edu (F.C.); i@xenobe.org (J.J.L.C.)

The authors wish to make the following correction to paper [1], doi:10.3390/molecules20023027, website: http://www.mdpi.com/1420-3049/20/2/3027/.

In Figure 3, the structures of NMS-859, ML240 and ML241 have been corrected. The molecules labeled NMS-873 and 2-(cyclohexylmethylamino)pyrimidines were mislabeled and have been corrected. The structure for alkylsulfanyl-1,2,4-triazoles has also been corrected.

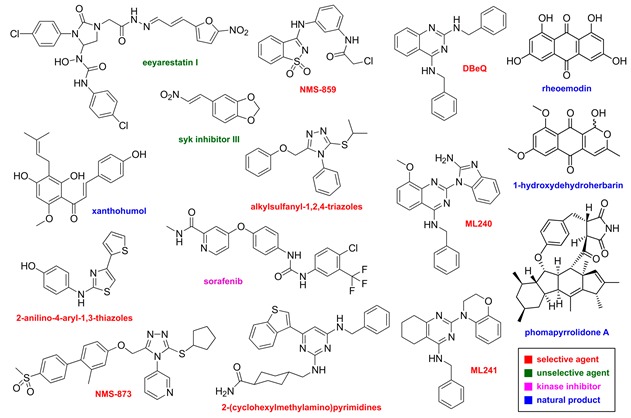
(1)


The authors would like to apologize for any inconvenience caused to the readers by these changes.
